# Effect of 24/7 attending coverage in the neonatal intensive care unit on fellow education

**DOI:** 10.1186/s12909-020-02372-2

**Published:** 2020-11-18

**Authors:** Mitali Sahni, Anja Mowes

**Affiliations:** 1 Neonatal Intensive Care Unit, Pediatrix Medical Group, Sunrise Children’s Hospital, 3186 S Maryland Pkwy, Las Vegas, NV 89109 USA; 2 University of Nevada, Las Vegas, NV USA; 3 Division of Neonatology, St. Christopher’s Hospital for Children, Philadelphia, PA USA

**Keywords:** Education, Supervision, Autonomy, Neonatology, House staff, Survey

## Abstract

**Background:**

There is a current change in type of attending coverage in the Neonatal Intensive Care Unit (NICU) from home calls to 24/7 in house coverage. Effects of this increased attending physician presence on education of NICU fellows has not been studied. The objective of this study is to evaluate the fellows’ perception of in house attending coverage on their education and evaluate its effect on their perceived autonomy.

**Methods:**

A secure, anonymous, web-based survey was designed using RedCap. The web-based survey was sent via the section of Neonatal Perinatal Medicine of the American Academy of Pediatrics, to all members of Training & Early Career Neonatologists. Questions were focused on perception of IH attending coverage on fellows’ educational experience including the respondent’s perceived ability to make independent decisions (autonomy). Chi-square tests were used to compare responses between groups, with Fisher Exact tests used when the expected cell frequencies were small.

**Results:**

One hundred and twenty-three surveys were analyzed, that included responses from 82 fellows & 41 early career neonatologists. 52% reported having 24/7 attending in-house (IH) coverage. Thirty of the 123 respondents experienced a change in model of attending coverage during their training. Among these 30, only 26.6% preferred the model of attending IH coverage. The respondents currently working in IH models, when compared to those in non-IH coverage models felt IH attending coverage was beneficial for fellow education (*p* < 0.05) but was less likely to give fellows autonomy for decision making (*p* = 0.02).

**Conclusion:**

In our survey respondents with in house attending, had a more favorable view of its benefit on fellow education. Institutions practicing or considering IH attending coverage should consider use of adequate measures to balance fellow supervision and education.

**Supplementary Information:**

The online version contains supplementary material available at 10.1186/s12909-020-02372-2.

## Background

There is a current change in type of attending coverage in the neonatal intensive care units (NICU) across the United States where more programs are transitioning from home coverage to 24/7 in house (IH) attending coverage. In the era of increased duty hour restrictions for house staff and stronger recommendations for their supervision by Accreditation Council for Graduate Medical Education (ACGME), the attending presence in the intensive care units (ICU) has increased [1]. At our institution we transitioned to attending in house coverage in July 2017. This change in practice has been attributed to various reasons including but not limited to improved patient outcomes, presence of additional help during emergent situations and improvement in practice [2]. However, the data available does not clearly support this thought and is, at best, ambivalent [3–5].

Some have argued that changing to an IH model will affect the fellows’ education in a negative way by decreasing their autonomy to make decisions [6]. Experts fear that in this model, fellows tend to defer to attending physicians to make decisions, instead of making and defending their own decisions [6]. This practice may also lead to younger attending physicians being less comfortable with allowing physicians in training to make clinical decisions on their own [6]. During a recent study done to evaluate the perception of 24/7 intensivist presence in the Pediatric Intensive Care Unit (PICU) on house staff education – a large number of respondents felt that the house staff was not prepared for independent practice after training in a IH attending coverage model [7]. Concerns about fellows’ autonomy were also raised in a study done to evaluate their education in Pediatric Cardiac Intensive Care unit [8]. Effects of increased attending physician presence on education of NICU fellows have not been studied. We hypothesized that 24/7 intensivists’ coverage in the NICU at academic institutions would cause a perception of decreased autonomy and would not be perceived as being beneficial for their education.

## Methods

We designed an anonymous web-based survey of 13 questions and distributed the survey by using the secure RedCAP database. This survey was sent via the section of Neonatal Perinatal Medicine of the American Academy of Pediatrics, to all members of Training & Early Career Neonatologists (TECaN) targeting the current fellows and recently graduated attendings that were part of the TECaN list serve. Questions in the survey were created by the study team based on their experience in NICU and with fellow education. Questions were focused on perception of IH attending coverage on fellows’ educational experience. The initial survey instrument was tested by a few fellows and early career neonatologists at the author’s parent institution and was revised based on their feedback. The results from this initial testing were not included in the survey results of the study.

IH coverage was defined as the presence of a neonatology attending in the hospital 24 h per day, 7 days a week, whereas home coverage (HC) was defined as neonatology attending taking calls from outside the hospital during nontraditional hours (nights and weekends). There were also some mixed coverage models reported where the attending stayed in house with new trainees or under discretionary circumstances. We used a 5-point Likert scale (strongly agree, agree, neutral, disagree, strongly disagree) to assess subjective questions regarding the respondents’ perception of the effect of attending presence in the NICU. To facilitate the assessment of the respondent’s perception, we grouped the strongly disagree and disagree as one group - “disagree” and similarly combined together strongly agree and agree as - “agree” while neutral formed the third group. A copy of the survey instrument used has been provided as supplement 1.

We compared the difference in perception between respondents in IH attending model versus (vs.) respondents from HC and mixed models. There was no specific definition used for “autonomy” in the study, since we evaluated the “perceived autonomy” by fellows and would be determined by the respondent’s perception and not by the investigator. Our sample size was determined by the number of responses and not by a power analysis. Given 64 IH and 59 non-IH respondents, doing a chi-square test on a yes/no outcome (such as agree/strongly agree versus all other responses), we would have 80% power, two-tailed, for a difference on the order of 50% versus 25% agree/strongly agree between the two groups. Chi-square tests were used to compare responses between groups, with Fisher Exact tests used when the expected cell frequencies were small. *P* values < 0.05 were considered significant. The institutional review board at Drexel University approved this study with a waiver of consent.

## Results

The survey was sent to 1041 subjects that were part of the TECaN list serve. However, not all members were eligible to take the survey, since some early career neonatologists had completed training more than 5 years ago. We report the results from 123 completed surveys that include responses from 82 fellows & 41 early career neonatologists that graduated within the past 5 years. 52% trained in NICUs with IH attending coverage. 36% trained in institutions with attending HC model and 12% were from a mixed model where the attending stayed in house in certain situations.

**Fig. 1:**
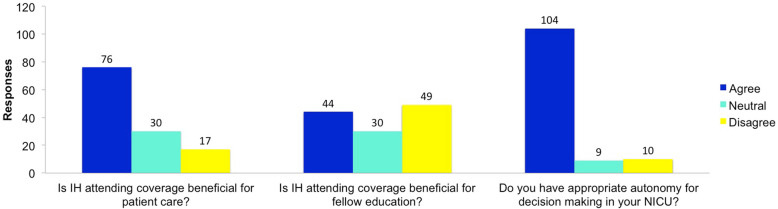
Fellows perception of in house attending coverage

Most participants perceived the effect of IH attending coverage on patient care to be beneficial (Fig. 1). However, when asked if the presence of attending in the NICU improved the fellow’s education the responses divided almost equally between agree, neutral, and disagree (Fig. 1). More people in the IH attending model than the HC and Mixed Model (54.6% versus 15.2%) felt that having attending in house was beneficial for their education, *p* < 0.05. The majority of respondents felt they had autonomy in their NICU (Fig. 1). However, more respondents in a model with IH attending disagreed with having autonomy, than those in HC and Mixed Model (10.9% versus 5%), *p* = 0.02.

Interestingly, 30 of the 123 respondents experienced a change in model of attending coverage during their fellowship training. Among these 30 respondents, only 28% preferred having the model of attending IH coverage, while 14% commented on the benefits of both models. For instance, one subject responded that his/her preference, “depended on the hospital- in an ECMO center, preferred in house attending call, while in a 70 bed Level III, preferred attending to be at home.” The ACGME encourages programs to have a checklist that provides fellows with guidelines to suggest - when to call attending with situations/patient status changes [9]. Only 30.9% of the respondents acknowledged having such a checklist.

Most respondents perceived the reason for the increased attending presence to be better patient safety and having more help in emergent situations. Our survey participants acknowledged that programs with attending IH took certain measures to promote fellows’ autonomy that have been listed in Table 1. When asked about the factors that most influenced the fellows’ autonomy, our participants perceived them to be attendings’ preferences and fellows’ experience. Another respondent expressed that the autonomy was also affected by the division practices. For example, the respondent noted that, “During residency their second-year resident supervised interns at meconium exposed deliveries. While as a fellow, they have an intern, resident, fellow and sometimes attending present at the delivery.” In their opinion, “It does not allow for autonomy and limits education. I feel it can be harmful after graduation when there is no longer someone physically present that is supervising you.”

**Table 1:** Fellows’ perception of IH attending coverage model

Question	Most frequent answers (number of respondents that chose the listed reason – could choose multiple reasons)
Most important reason for switching to IH model	-Patient safety (37/123, 30.1%) -Possible emergency situation that requires additional help (37/123, 30.1%)
In IH model, how does the program encourage fellow’s autonomy	-Fellows encouraged to act independently/make a plan prior to consulting attending (79/123, 64.2%) -Nurses encouraged to call fellow first (68/123, 55.3%)
Factor influencing fellow’s autonomy for decision making	- Attending’s preference (103/123, 83.7%) - Fellow’s experience (98/123, 79.7%) - Patient pathology (87/123, 70.7%) - Attending’s age (49/123, 39.8%)

## Discussion

There has been an ongoing debate about the effect of 24/7 intensivist coverage in critical care units in both adult and pediatric hospitals [10]. Numerous studies have been done in the PICU and pediatric cardiac ICUs to evaluate the effect of this on patient outcomes and house staff education [7, 11]. However there is a paucity of such investigations in the NICU and the published data is not very reliable. In our study we received responses from 123 fellows and early career neonatologist from 1041 subjects. Since some early career neonatologists had completed training more than 5 years ago, they were not eligible to take the survey and hence, our response rate of 11.8% is not accurate. We chose to compare the results of the IH group with the non-IH group, in which we combined the responses from HC and mixed model groups. Since, the mixed models were primarily home coverage and only did IH coverage in specific situations, we believe these groups were better placed together. This also allowed us to better perceive the opinion about IH coverage from respondents that only worked in that model vs. those that did not. The results from our survey suggest that most respondents perceive that concerns about patient care and safety have led to an increase in IH coverage. There are conflicting feelings regarding the benefits of IH coverage on fellow education, however more participants in the IH coverage model perceived it to be better for fellow education than participants in non-IH models.

In a previous study done to evaluate patient volume, staffing and workload in relation to risk adjusted outcomes; the UK Neonatal Staffing Study Group reported less nosocomial infections and quantitatively less death or brain damage with less neonatal consultant coverage [5]. In this study, the authors defined neonatal consultant coverage as pediatricians with more than 50% of their clinical sessions committed to neonatal care. In another study done in Canadian NICUs the investigators showed that units with in-house faculty or fellow coverage had lower nocturnal mortality rates relative to units with coverage by residents or other personnel [12]. Despite this unclear data, there is a vast majority of academic centers with fellows in training that are adapting the IH attending coverage model.

In a large observational study done using a virtual Pediatrics System Database, Gupta et al. demonstrated that 24/7 IH attending coverage in the PICU is associated with improved overall patient care and survival after cardiac arrest compared with ICU’s with HC model [4]. In a national survey of pediatric intensivists, pediatric critical care fellows and residents evaluating the perception of 24/7 IH attending on house staff education, only 50% of intensivists and 67% of house staff felt that house staff was well prepared for independent practice after training in an IH model of attending coverage. In this survey respondents currently working in IH models had a more favorable perceptions of the effects of IH coverage on house staff autonomy (*P* < .0001), supervision (*P* < .0001), and preparation for independent practice (*P* < .0001) when compared with those training in HC models [7]. Similarly, in our survey respondents at institutions with IH coverage felt strongly that IH coverage was beneficial for fellows’ education when compared to respondents from institutions with HC and mixed model. This may be due to the respondent’s familiarity with their model or that centers with IH model may have found ways to adapt to this changed model of attending coverage and utilize attending presence for improving the fellows educational experience. This may also suggest that there is a biased perception amongst respondents from institutions with HC model about the deleterious effects of IH coverage on fellows’ education.

The current guidelines of increased supervision requirement by ACGME [13] and recommendation of the society of Critical Care Medicine to have an intensivist led care of patients in the ICU have led to more institutes moving towards increased attending in house presence [14]. This increased attending presence in the NICU could provide an opportunity to improve fellows’ education. Another suggestion would be to encourage fellows to have a plan of action ready prior to consulting attendings and attendings could utilize this opportunity to convert the bedside clinical situation into a teachable moment.

There is a delicate balance between supervision and autonomy. In our survey, only 10 respondents felt that they did not have autonomy in their NICU. The fellows that felt lack of autonomy in their NICU were more likely to be training in centers with IH attending coverage. 83.7% of our respondents noted attending preference and 39.8% noted attending’s age as factors influencing the level of autonomy, they get in their NICU. In a commentary about increased attending presence in the NICU, Jobe and Martin remarked, “Younger attending physicians may be less comfortable in allowing physicians-in-training to make decisions, manage patients, and develop independence. Residents then become scribes for the clinical team rather than active participants, and fellows defer to attending physicians rather than making and defending decisions.” [6].

Fellowship programs have come up with different strategies to help provide fellows autonomy in the presence of IH attending. The most frequent strategies on how their program promoted fellows autonomy, involved: encouraging nurses to call the fellows first and encouraging fellows to make their own plan before calling the attending. Another suggestion could be for the attending to not be physically present in the unit during call. This would decrease the chances of the nurses approaching the attending with concerns directly and allowing them to be available promptly when the fellow needs help. In interest of patient safety, the ACGME recommends that fellowship program should have a checklist that provides fellows with guidelines for circumstances and events in which fellows must communicate with their supervising faculty [9]. Only 30.9% of our respondents acknowledged having such a checklist. A better use of this tool may also help with addressing the issue of patient safety and balancing autonomy.

Although our study provides interesting and enlightening data, it is limited by the survey design and route of distribution. Since the TECaN list serve does not have a separate list for current fellows and early career neonatologists, we are unable to provide a response rate for current fellows who took this survey. Another limitation is the low response rate of the survey and may affect the validity of the results. The study also suffers from reporting bias since it is a self-reported survey and our ability to interpret the data. In addition this study only provides data about the perception of the respondents to change in attending coverage model. It does not objectively determine its effect on fellow competence, patient care and patient outcomes. However, it helps identify the major areas of concern with the perception of IH attending coverage in the NICU and can help guide programs to better address these concerns and implement measures to improve fellows’ educational experience. Based on the results from this survey a qualitative follow up study could be done, focusing on the fellows in HC model and analyze why they feel that attending presence would not be beneficial.

## Conclusion

Concern for patient care and safety have appropriately led to increased attending presence in the NICU. However, its impact on fellow education and autonomy are important considerations that have not been well studied. Our survey indicates that there are conflicting feelings regarding its benefit on training of neonatal fellows, however fellows training in IH model find it to be beneficial for their education. The centers undergoing this transformation should consider taking adequate measures to balance patient safety and fellow’s autonomy as well as utilize the increased attending presence as a tool to improve fellows’ educational experience.

## Supplementary Information

**Additional file 1.**

## Data Availability

The datasets used and/or analyzed during the current study are available from the corresponding author upon reasonable request.

## Abbreviations

NICU: Neonatal Intensive Care Unit
IH: In house
ACGME: Accreditation Council for Graduate Medical Education
ICU: Intensive care units
PICU: Pediatric Intensive Care Unit
TECaN: Training & Early Career Neonatologists
HC: Home coverage
