# Supplementary material for: Effect of 24/7 attending coverage in the neonatal intensive care unit on fellow education
Source: BMC Med Educ. 2020 Nov 18;20:444. doi: 10.1186/s12909-020-02372-2 (PMC7672982; doi:10.1186/s12909-020-02372-2)
Supplement: Supplementary file 1 — Additional file 1. [file 12909_2020_2372_MOESM1_ESM.pdf]

Effect of 24/7 attending coverage in the Neonatal Intensive Care Unit on fellow education

Please complete the survey below.

Thank you!

What is you current level of training?
- ◯ PGY 4
- ◯ PGY 5
- ◯ PGY 6

What is the current model of fellow night-time coverage in your program?
- ◯ Fellow 24/7
- ◯ Fellow on call from home/NNP or PA in house
- ◯ Fellow on call from home/resident in house
- ◯ Other

What is the current model of attending night-time coverage in your program?
- ◯ Attending in house 24/7
- ◯ Fellow in house/attending on call from home
- ◯ Mixed model: attending in house when new/inexperienced fellow on call, and attending on call from home when more advanced fellow in house
- ◯ Fellow and attending on call from home

What would be the most important reason for you behind transitioning to an in-house attending coverage mode?
- ◯ Improvement in supervision and teaching
- ◯ Patient safety
- ◯ Possible emergency situation that require additional help
- ◯ Institutional policy
- ◯ Improved comfort level

If your attending are in house overnight, how does the program seeks to encourage independence for the fellow's decision making? (Check all that applies)
- ☐ Attending is not physically in the unit
- ☐ Attending is encouraged not to participate in night rounds
- ☐ Nurses are encouraged to call fellow first
- ☐ Fellows are encouraged to handle situations independently or make a plan of action prior to consulting the attending
- ☐ Other

Please describe ________________________________

Does your program provide a checklist with situations/patient status changes as guideline when to call the attending?
- ◯ Yes
- ◯ No

If not, would such a checklist be helpful for you?
- ◯ Yes
- ◯ No

Has the model of attending night-time coverage in your program changed during the period of your training from attending coverage from home to attending being in house?
- ◯ Yes
- ◯ No

If yes, which model do you prefer? ________________________________

Do you feel having an attending in house is beneficial for patient care?
- ◯ Strongly agree
- ◯ Agree
- ◯ Neutral
- ◯ Disagree
- ◯ Strongly Disagree

Do you feel having an attending in house is beneficial for your education as a neonatology fellow?	◯ Strongly agree<br>◯ Agree<br>◯ Neutral<br>◯ Disagree<br>◯ Strongly Disagree
At the level of your current training do you feel comfortable managing nearly all patients in the NICU?	◯ Strongly agree<br>◯ Agree<br>◯ Neutral<br>◯ Disagree<br>◯ Strongly Disagree
Do you feel you have appropriate autonomy for decisions making in your NICU?	◯ Strongly agree<br>◯ Agree<br>◯ Neutral<br>◯ Disagree<br>◯ Strongly Disagree
What factors influence the level of autonomy you get in the NICU for patient care? (check all that applies)	☐ Patient pathology<br>☐ Attending's age<br>☐ Attending's preference<br>☐ Fellow's experience<br>☐ Other
Please describe	__________________________________
